# Blap-6, a Novel Antifungal Peptide from the Chinese Medicinal Beetle *Blaps rhynchopetera* against *Cryptococcus neoformans*

**DOI:** 10.3390/ijms25105336

**Published:** 2024-05-14

**Authors:** La-Mei Zhang, Sheng-Wen Zhou, Xiao-Shan Huang, Yi-Fan Chen, James Mwangi, Ya-Qun Fang, Ting Du, Min Zhao, Lei Shi, Qiu-Min Lu

**Affiliations:** 1 Institute of Highland Forest Science, Chinese Academy of Forestry, Kunming 650224, China; 18855038552@163.com (L.-M.Z.); duting_x_y@163.com (T.D.); mzhao@caf.ac.cn (M.Z.); 2 Key Laboratory of Breeding and Utilization of Resource Insects, National Forestry and Grassland Administration, Kunming 650224, China; 3 Engineering Laboratory of Peptides of Chinese Academy of Sciences, Key Laboratory of Bioactive Peptides of Yunnan Province, KIZ-CUHK Joint Laboratory of Bioresources and Molecular Research in Common Diseases, National Resource Center for Non-Human Primates, National Research Facility for Phenotypic & Genetic Analysis of Model Animals (Primate Facility), Key Laboratory of Genetic Evolution & Animal Models, Sino-African Joint Research Center, and New Cornerstone Science Laboratory, Kunming Institute of Zoology, the Chinese Academy of Sciences, No.17 Longxin Road, Kunming 650201, China; zhoushengwen@mail.kiz.ac.cn (S.-W.Z.); huangxiaoshan@mail.kiz.ac.cn (X.-S.H.); chenyifan@mail.kiz.ac.cn (Y.-F.C.); jams@mail.kiz.ac.cn (J.M.); fangyaqun@mail.kiz.ac.cn (Y.-Q.F.); 4 Kunming College of Life Science, University of Chinese Academy of Sciences, Beijing 100049, China

**Keywords:** *Cryptococcus neoformans*, blap-6, *Blaps rhynchopetera*, antimicrobial peptide

## Abstract

*Cryptococcus neoformans* (*C. neoformans*) is a pathogenic fungus that can cause life-threatening meningitis, particularly in individuals with compromised immune systems. The current standard treatment involves the combination of amphotericin B and azole drugs, but this regimen often leads to inevitable toxicity in patients. Therefore, there is an urgent need to develop new antifungal drugs with improved safety profiles. We screened antimicrobial peptides from the hemolymph transcriptome of *Blaps rhynchopetera* (*B. rhynchopetera*), a folk Chinese medicine. We found an antimicrobial peptide named blap-6 that exhibited potent activity against bacteria and fungi. Blap-6 is composed of 17 amino acids (KRCRFRIYRWGFPRRRF), and it has excellent antifungal activity against *C. neoformans*, with a minimum inhibitory concentration (MIC) of 0.81 μM. Blap-6 exhibits strong antifungal kinetic characteristics. Mechanistic studies revealed that blap-6 exerts its antifungal activity by penetrating and disrupting the integrity of the fungal cell membrane. In addition to its direct antifungal effect, blap-6 showed strong biofilm inhibition and scavenging activity. Notably, the peptide exhibited low hemolytic and cytotoxicity to human cells and may be a potential candidate antimicrobial drug for fungal infection caused by *C. neoformans*.

## 1. Introduction

Fungal infections have led to an increase in patients and fatalities over the past several years [1]. *Cryptococcus neoformans* is an opportunistic fungal pathogen widely distributed in the natural environment. It poses a serious concern for public health and safety due to its high morbidity and mortality in individuals with inadequate immunity [2,3]. After inhaling yeast cells or spores, asymptomatic infection occurs in the lungs of humans, and symptoms develop as the host’s immune system deteriorates [4,5]. In extreme cases, it can infiltrate the central nervous system and cause cryptococcal meningitis. With a high mortality rate, the fungus is responsible for nearly 1 million incident cases and about 200,000 deaths per year worldwide [6,7,8,9,10]. Currently, the clinical drugs for the treatment of *Cryptococcus neoformans* (*C. neoformans*) infection mainly include azole drugs, amphotericin B, and 4-fluorocytosine [11]. Due to the side effects of amphotericin B and 4-fluorocytosine on humans, including kidney damage, and the increase in the number of clinically resistant isolates, it is urgent to develop alternative antifungal drugs for *C. neoformans* infection. Furthermore, antibiotics are harmful to the environment, quickly produce drug-fast growth in *C. neoformans*, and can quickly lead to the development of more severe and unpredictable issues in the future from disease-causing pathogens [12].

During the formation of microbial infections and wounds, insects release a family of tiny molecular polypeptides known as insect antimicrobial peptides (AMPs), which have antibacterial properties. They are crucial defense mechanisms in the innate immune system of insects and effectively eliminate bacteria, fungi, and viruses [13,14]. For example, a large number of AMP genes have been found in *Drosophila*, honeybees, and silkworms [15,16,17]. Insect AMPs primarily eliminate pathogenic microorganisms by compromising the integrity of cell membranes. They also partially attach to bacterial nucleic acids to inhibit their biosynthetic function and show antibacterial properties [18]. The majority of AMPs are cationic AMPs that contribute to changes in membrane permeability [19]. By interacting with the anions on the surface of pathogenic microorganisms, AMPs are adsorbed to the surface of microbial cell membranes. At the same moment, their hydrophobic tails are introduced into the hydrophobic area of the cell membrane [20,21]. Antimicrobial peptide molecules gradually build ion channels on the cell membrane as they accumulate, alter the structure of the cell membrane, damage the cell membrane, cause intracellular material leakage, and ultimately kill pathogenic bacteria [22]. Many insect antifungal peptides exhibit broad-spectrum antifungal action with low drug resistance and few toxic and adverse consequences [19]. 

Cysteines carry information related to the secondary structure of AMPs, and their positions and pairing motifs can provide identifiable information [23]. According to their secondary structure classification, they are divided into: (1) Linear α-helical peptides without cysteine, such as cecropin A from *Plutella xylostella* [24]; (2) β-sheet peptides containing one or more disulfide bonds, such as thanatin from *Podisus maculiventris* [25]; (3) Linear peptides without α-helix and β-sheet structures but usually rich in specific amino acids (including Gly, Arg, Trp, or Pro), such as apidaecins from *Apis mellifera* and attacins from *Hyalophora cecropia* [26,27]; (4) Peptides containing both α-helix and β-sheet structures, such as insect defensins; they all contain a common CSαβ motif [28]. Our study will explore the structure of the newly discovered antimicrobial peptides.

Medicinal insects are one of the best sources for the development of new drugs, and they belong to a type of animal medicine widely used in traditional Chinese medicine [29]. *Blaps rhynchopetera* (*B. rhynchopetera*, Coleoptera, Tenebrionidae) is a species of folk Chinese medicinal insect [30]. It is popular as a medicine due to its high therapeutic value, which includes considerable hypoglycemic, antibacterial, anti-tumor, anti-inflammatory, and other activities [31]. Although *B. rhynchopetera* has great potential for use in medicine, research on its active ingredients and efficacy verification is very limited, resulting in a limited understanding of its effective medicinal substances and active mechanisms [32]. In recent research, *B. rhynchopetera* was shown to contain blapstin, a new anti-fungi peptide that is effective against *Candida albicans* and *Trichophyton rubrum* [33]. Based on this, we identified a novel peptide called blap-6 from the hemolymph transcriptome of *B. rhynchopetera*, which is effective against bacteria and fungi, especially *C. neoformans*. In addition, the potential mechanism of blap-6’s antifungal activity against *C. neoformans* was studied. Blap-6 may be a candidate for the development of a new antimicrobial peptide drug against *C. neoformans* infection.

## 2. Results

### 2.1. Physicochemical Property

In recent years, short peptides have attracted much attention due to their high diversity and extensive bioactivity spectra [34]. Tbtools software (version Tbtools v2.056) was used to filter the positively charged amino acids in the hemolymph transcriptome of *B. rhynchopetera*, and 11 peptides (<40 amino acids) with antibacterial activity were predicted by the deep learning method. These predicted peptides are all short peptides with amino acid lengths of 15–35 and are easy to synthesize. And then, we used the ExPASy website to analyze the hydrophilicity and hydrophobicity of these peptides. The results showed that there were five hydrophilic peptides and six hydrophobic peptides (Table 1).

### 2.2. Hydrophobicity and Structure of Blap-6

**Figure 1:**
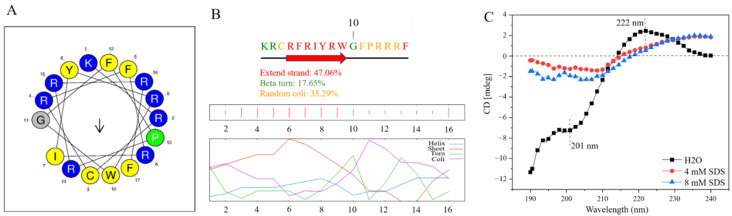
The hydrophobicity and structure of blap-6. (**A**) The helix wheel of blap-6 was analyzed using the HeliQuest website, the red ‘1’ represents the starting position and the arrow represents hydrophobic moment. (**B**) The secondary structure of blap-6 was predicted using the secondary structure analysis software on the NPS@ website (https://npsa-prabi.ibcp.fr/cgi-bin/npsa_automat.pl?page=/NPSA/npsa_sopma.html, accessed on 11 May 2024). (**C**) The conformational changes in blap-6 in different solvent environments. Spectra were the accumulation of three scans measured in 0 mM, 4 mM, and 8 mM SDS.

The hydrophobicity of peptides is one of the factors used to predict whether they have antibacterial activity [35]. To further understand the hydrophobic properties of blap-6, we used HeliQuest website to analyze it and found that the polar and non-polar amino acids of blap-6 were evenly distributed. The sequence of blap-6 has nine polar amino acids (52.94%) and eight non-polar amino acids (47.06%). The values of average hydrophobicity and average hydrophobic moment are 0.269 and 0.234, respectively (Figure 1A). These calculated results indicate that blap-6 may have an amphiphilic structure, which may contribute to its antibacterial properties.

The secondary structure of proteins depends on the orderly arrangement of peptide bonds, which is mainly maintained by hydrogen bonds between molecules. The protein buffer system can affect the hydrogen bond interaction and lead to changes in the secondary structure. We used the NPS@ website to predict the two-dimensional structure of blap-6. The results show that the β-sheet of blap-6 accounts for 47.06%, the β-turn accounts for 17.65%, and random coli accounts for 35.29% (Figure 1B). To verify the NPS@ website predictions, we performed CD (Circular Dichroism) detection on blap-6 in different SDS buffers. We found that the characteristics of blap-6 CD spectra dissolved in different SDS were significantly different. The CD spectra of blap-6 dissolved in 0 mM SDS were more obvious, showing a negative peak near 201 nm and a broad positive peak near 222 nm, indicating a secondary structure dominated by β-sheet and random coil, forming an αβ motif. The fitting results show that the relative contents of α-helix, β-sheet, β-turn, and random coil were 5.0%, 43.3%, 11.2%, and 40.5%, respectively. These results indicate that the secondary structure of the protein in the aqueous solution system was mainly β-sheet and random coli, forming an αβ motif with an α-helix and a β-sheet. The CD spectra of blap-6 dissolved in 4 mM and 8 mM SDS were similar, indicating that the effect of different concentrations of SDS buffer on the protein is not obvious. However, compared with 0 mM SDS, the absorption peaks of the two samples shifted, and the absolute intensity of the absorption peaks at 201 nm and 222 nm decreased, suggesting that SDS buffer can affect the optical activity of the protein and disrupt its secondary structure (Figure 1C). The fitting results also show a slight increase in the relative content of α-helix and a slight decrease in the relative content of β-sheet structure in the SDS buffers. The above conclusions indicate that the secondary structure of blap-6 is affected by the buffer system, with the SDS buffer having a more pronounced effect. Although there are some differences between the NPS@ website prediction and the CD test results, we take the result of the CD test as the criterion for determining the structure of the peptide.

### 2.3. Sequence Alignment and Phylogenetic Analysis

**Figure 2:**
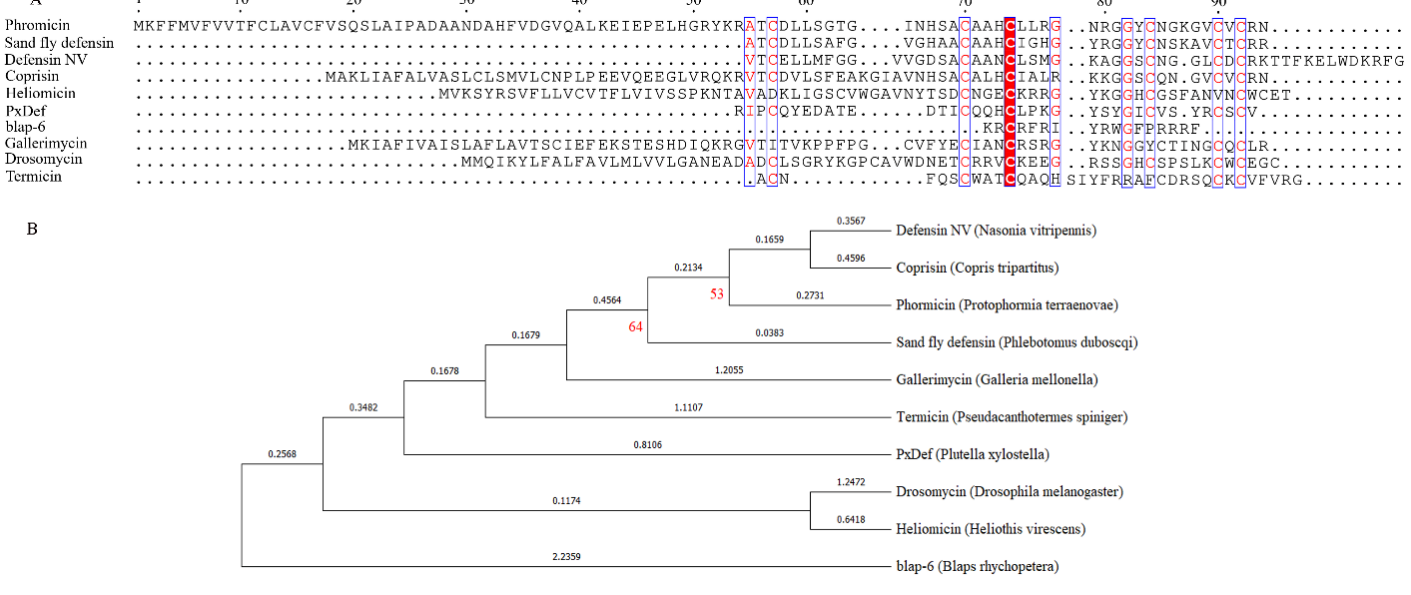
Sequence alignment and phylogenetic analysis for blap-6 and the insect defensin family. (**A**) Sequence alignment for blap-6 and insect defensin families by Clustal W, the box and the color represents amino acid identity of these sequences. (**B**) phylogenetic analysis for blap-6 and insect defensin families by Mega software. The red number represents the reliability of the branch. When the value is less than 50, it is hidden. The black number represents the length of the branch. The larger the value, the farther the branch. Defensin NV, insect defensin from *Nasnia vitripennis* [40]; coprisin (Genebank: A9XFZ7), insect defensin from *Copris tripartitus* [41]; Phormicin (Genebank: P10891), insect defensin from *Protophormia terraenovae* [42]; Sandfly defensin, insect defensin from *Phlebotomus duboscqi* [43]; Gallerimycin (Genebank: Q8MVY9), insect defensin from *Galleria mellonella* [44]; Termicin (Genebank: P82321), insect defensin from *Pseudacanthotermes spiniger* [37]; PxDef, insect defensin from *Plutella xylostella* [45]; Drosomycin (Genebank: P41964), insect defensin from *Drosophila melanogaster* [39]; Heliomicin (Genebank: P81544), insect defensin from *Heliothis virescens* [38].

The Basic Local Alignment Search Tool (Blast) website is for alignment homology searches, and no sequence similar to blap-6 was found. Structural analysis reveals that blap-6 has an αβ motif, although it has only one cysteine, which is significantly different from antifungal peptides from the insect defensin family. Insect defensins generally contain 34–46 amino acid residues with a positive charge, which are the most famous peptides with CSαβ motifs. They all have C-CXXXC-C-CXC consensus sequences and contain 3–4 pairs of intramolecular disulfide bonds [36]. Their structure includes an α-helix connected to a double-stranded β-sheet to form an αββ motif, such as termicin from *Pseudacanthothermes spiniger* [37], or connected to a three-stranded β-sheet to form a βαββ motif, for example, heliomicin from *Heliothis virescens* and drosomycin from *Drosophilia melanogaster* [38,39]. Therefore, we compared the sequence of blap-6 with the insect defensin family and found that the cysteine of blap-6 is in the same position as the insect defensin family, indicating that it may form a β-sheet structure (Figure 2A). In order to trace the relationship between blap-6 and insect defensins, we conducted a phylogenetic analysis. The results showed that although blap-6 was located on the same branch as the antifungal peptide heliomicin, the confidence of this group was low, and each branch was very long (Figure 2B). Therefore, the two peptides are likely to be distantly related. From these results, we conclude that blap-6 is a novel peptide with a cysteine-stabilized αβ motif whose homology is far from insect defensins.

### 2.4. Antimicrobial Activity of Predicted Peptides

We used a double dilution method to determine whether these predicted peptides have antibacterial activity against these selected strains. The results show that except for blap-1, blap-2, blap-3, blap-4, blap-5, and blap-11, these predicted peptides exhibit antibacterial action, including blap-6, blap-7, blap-8, blap-9, and blap-10. Blap-6 is active against both bacteria and fungi, and it is more potent against fungi. The MIC of blap-6 against *C. albicans* is 1.22 μM. Importantly, the MIC of blap-6 again, *C. neoformans*, is 0.81 μM, which is 6-8 times lower than that of fluconazole. However, it was 15 times higher than that of amphotericin B (Table 2).

**Figure 3:**
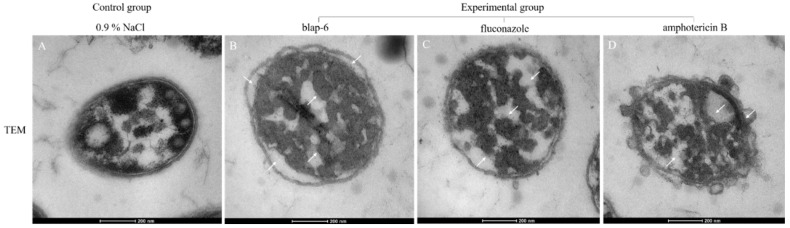
Transmission electron micrographs (TEM) of *C. neoformans* cells treated with blap-6. (**A**) Untreated *C. neoformans* showed a normal plasma membrane and an intact cell wall. (**B**) After treatment with 1 × MIC of blap-6. (**C**) After treatment with 1 × MIC of fluconazole. (**D**) After treatment with 1 × MIC of amphotericin B. Compared with the normal cells, treated *C. neoformans* exhibited morphological alterations, including cell wall thinning and cell membrane collapse (the white arrows refer). The control group was treated with sterile 0.9% NaCl.

The morphological differences between the untreated and blap-6-treated *C. neoformans* ATCC 32045 strains were examined using transmission electron microscopy (TEM) to determine the antifungal mechanism of the peptide. The findings revealed considerable differences. TEM analysis revealed that the untreated fungal cells had a thick, intact cell wall with clearly visible internal structures (Figure 3A). However, the peptide-treated cells exhibited severe disruption of the structural and cellular morphology—the cell membrane had deteriorated, the cell had swollen, the cell wall had thinned and separated from the membrane, the cell wall protruded outward, the vacuole had collapsed, the plasma membrane had disintegrated, and the cytoplasmic content had solidified (Figure 3B). These findings suggest that the antifungal activity of blap-6 against *C. neoformans* ATCC 32045 is mediated through disruption of the cell membrane and cell wall integrity, leading to the death of the fungal cells.

### 2.5. Killing Kinetics of Blap-6 against C. neoformans

**Figure 4:**
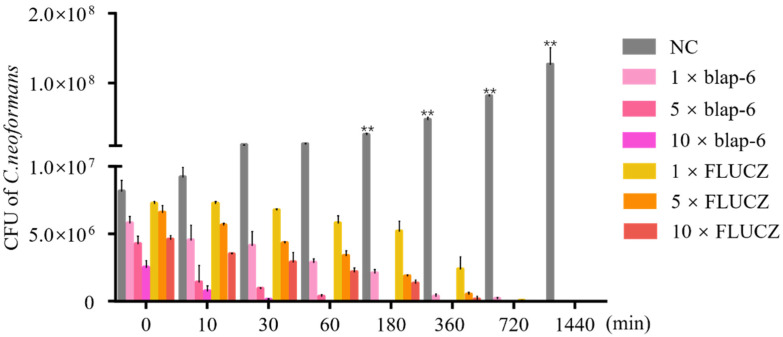
Fungal kinetics of blap-6 against *C. neoformans* ATCC 32045. FlUCZ was used for the positive control; the negative control is represented by sterile 0.9% NaCl. Abbreviation: NC, the negative control; FLUCZ, fluconazole. The results are expressed as the mean ± SD of three independent experiments. A two-way ANOVA was performed; **, *p* < 0.01.

Fungicidal kinetic assays can determine the in vitro fungal growth rate and directly measure the in vitro fungicidal activity of antimicrobial drugs. We measured its fungicidal rate to evaluate the antifungal effect of blap-6 against *C. neoformans* ATCC 32045. The results show that, compared with the positive control fluconazole, blap-6 had a more rapid and effective fungicidal effect, and the fungicidal rate was dose-dependent. The fungicidal rate of blap-6 against *C. neoformans* ATCC 32045 reached more than 90% within 180 min at a concentration of 5 × MIC, and all the fungi could be killed within 30 min at a concentration of 10 × MIC, whereas the fungicidal rate of fluconazole against *C. neoformans* ATCC 32045 required 360 min to reach more than 90% for the fungi to be completely killed (Figure 4). Therefore, blap-6 has more rapid fungicidal activity against *C. neoformans*.

### 2.6. Effects of Blap-6 on Biofilm

**Figure 5:**
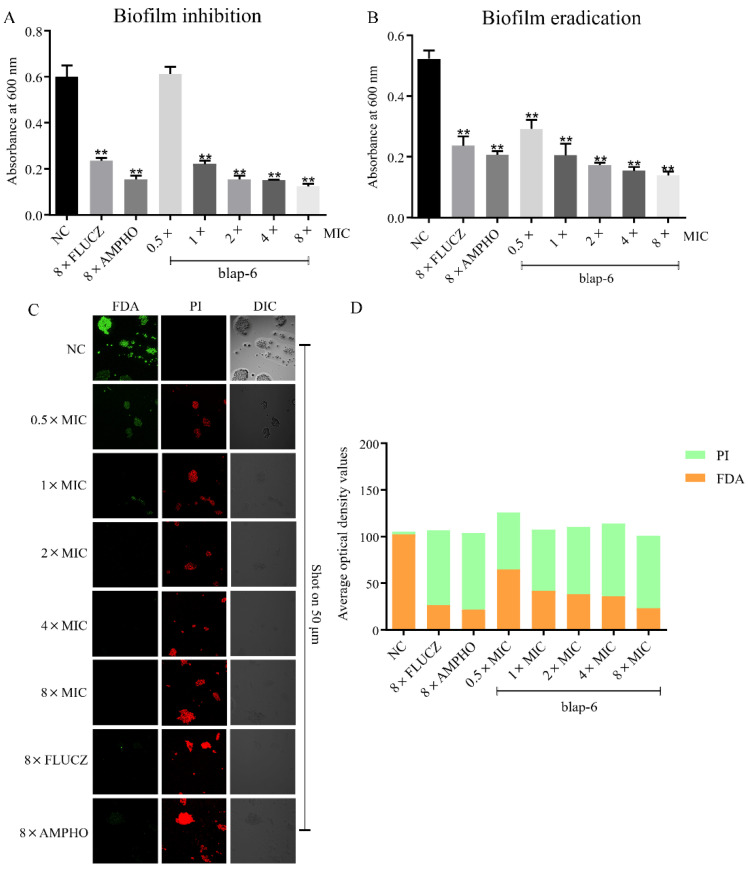
Effects of Blap-6 on Biofilm. (**A**) Blap-6’s inhibitory effects on the biofilm formation of *C. neoformans* ATCC 32045. (**B**) Blap-6’s effect on eradicating established biofilms of *C. neoformans* ATCC 32045. (**C**) Evaluation of blap-6’s effect on *C. neoformans* ATCC 32045 biofilms using a Two-photon laser scanning microscope (TPLSM, A1MP+, Nikon, Japan). Differential interference contrast (DIC) imaging was performed in white light. FDA-labeled cells were represented by green fluorescence, while PI-labeled cells were represented by red fluorescence. (**D**) Calculation of green or red fluorescence intensity using Image J (version Image J 2.15.0). The negative control (NC) was treated with 0.9% NaCl. Abbreviations: FLUCZ for fluconazole; AMPHO for amphotericin B. Results are presented as the mean ± SD of three independent experiments. A one-way ANOVA was performed; **, *p* < 0.01.

Biofilms are a fixed community of microorganisms embedded in an extracellular matrix that can attach to biological and non-biological surfaces. The existence of biofilms enhances the ability of microorganisms to resist multiple scavenging mechanisms, making them more difficult to eradicate. Our results demonstrate that blap-6 significantly inhibited the formation of *C. neoformans* biofilm at 1 × MIC (Figure 5A). Moreover, the ability of blap-6 to prevent biofilm growth becomes stronger as the concentration increases, indicating a dose-dependent effect. Similarly, in the biofilm eradication experiment, blap-6 exhibited the ability to remove pre-formed *C. neoformans* biofilm at 1 × MIC (Figure 5B). Further analysis using two-photon laser scanning microscopy (TPLSM) revealed a reduction in both the quantity and fluorescence intensity of FDA-labeled viable cells with increasing concentrations of blap-6. In contrast, the number and fluorescence intensity of PI-labeled non-viable cells exhibited a gradual rise. Specifically, the application of 8 × MIC of blap-6 demonstrated the most pronounced impact on the biofilm activity of *C. neoformans* ATCC 32045. Visual inspection under white light further corroborated these findings, showing that the *C. neoformans* ATCC 32045 biofilms treated with blap-6 appeared diminished and darker compared to the untreated control group (Figure 5C,D). Collectively, our results indicate that blap-6 has the potential to both inhibit the formation and eradicate pre-existing *C. neoformans* biofilms, highlighting its promising therapeutic potential against this fungal pathogen.

### 2.7. Mechanism of Blap-6 in C. neoformans Biofilm

**Figure 6:**
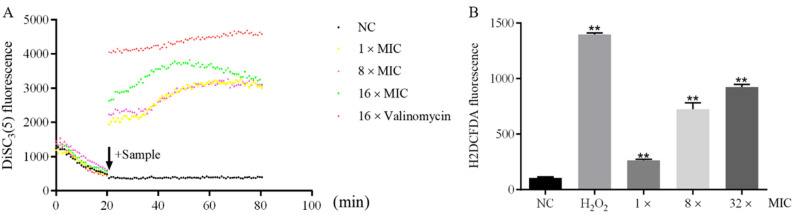
Possible mechanism of blap-6 on the biofilm of *C. neoformans* ATCC 32045. (**A**) DiSC3(5) staining monitors cell membrane potential changes. (**B**) H2DCFDA staining monitors the ROS burst. The NC was represented by 0.9% NaCl. H_2_O_2_ was referred to as 1 mM H_2_O_2_. The data represent the mean ± SD of three independent experiments. A one-way ANOVA was performed; **, *p* < 0.01.

To evaluate the effects of blap-6 on the cell membrane of Cryptococcus neoformans ATCC 32045, we analyzed changes in membrane potential using the fluorescent dye DiSC3(5). The results showed a significant increase in fluorescence intensity compared to the negative control, indicating disruption of the cell membrane. Treatment with 1 × MIC, 8 × MIC, and 16 × MIC of blap-6 led to an increased negative value in membrane potential and deviations toward cell hyperpolarization in a dose-dependent manner. However, the impact of blap-6 on the membrane potential of the fungi was not identical to that of the positive control, valinomycin (Figure 6A). Further investigation revealed that the production of reactive oxygen species (ROS), which are partly associated with cellular mitochondria [46], exhibited a concentration-dependent increase in *C. neoformans,* as indicated by enhanced fluorescence intensity (Figure 6B). This suggests that blap-6 may induce mitochondrial damage in *C. neoformans* to a certain degree.

### 2.8. Hemolysis and Cytotoxicity Assays

**Figure 7:**
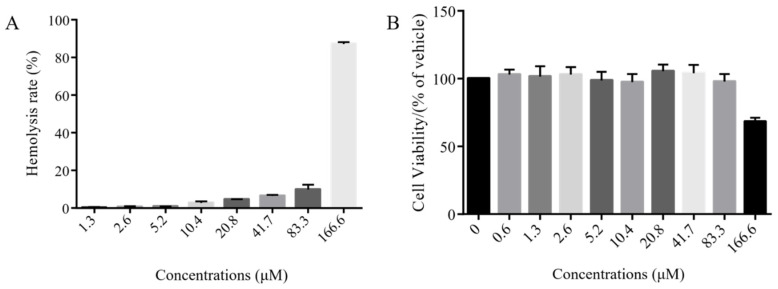
The hemolytic activity of blap-6 on human red blood cells and its cytotoxicity on mouse macrophages Raw 264.7 cells. (**A**) Hemolysis rate of blap-6 in human red blood cells. (**B**) Cytotoxicity of blap-6 on human HEK293 embryonic cells. NC is represented by sterile saline without blap-6. The results are expressed as the mean ± SD of three independent experiments.

Many antimicrobial peptides have excellent antibacterial activity, but those antimicrobial peptides also show strong hemolytic activity and cytotoxicity. Therefore, it is necessary to evaluate the hemolytic and cytotoxic activity of AMPs. In this experiment, the cytotoxicity of the polypeptide blap-6 to human HEK293 embryonic cells and the hemolytic activity of human red blood cells were tested. The experimental results are shown in Figure 7. Compared with the positive control, Triton X-100, the blap-6 had almost no hemolytic activity between 1.3 and 83.3 μM. The above results showed that blap-6 also had good safety in a certain concentration range, which laid foundation for its application to animal experiments (Figure 7A). In the concentration range of 41.7 μM, the survival rate of human HEK293 embryonic cells is above 100%. When the concentration was 83.3 μM, the cell survival rate was above 90%. When the concentration reaches 166.6 μM, the cell survival rate decreases, indicating that the peptide of blap-6 is toxic to mouse macrophages. Considering that the MIC of blap-6 was 0.81 μM, the antifungal effect of blap-6 can be guaranteed within the safe concentration range (Figure 7B).

### 2.9. Plasma and Protease Stability

**Figure 8:**
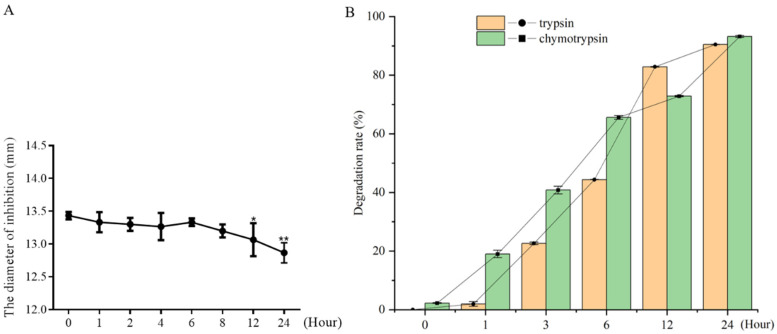
Stability of blap-6 in human plasma and proteases. (**A**) The antifungal activity of blap-6 against *C. neoformans* ATCC 32045 was determined by the disc diffusion method after incubation with human plasma for 0–24 h. (**B**) The effect of proteases (including trypsin and chymotrypsin) on the antifungal activity of blap-6. Data represent the mean ± SD of three independent experiments. One-way ANOVA and Two-way ANOVA were performed; *, *p* < 0.05, **, *p* < 0.01.

The plasma stability of a drug is an important factor that influences its dose, administration interval, and therapeutic efficacy. Drugs with good plasma stability tend to maintain their desired effects for extended periods and more consistently. To evaluate the plasma stability of blap-6, we examined its antifungal activity against *C. neoformans* after over-time incubation in plasma. As shown in Figure 8, the antifungal potency of blap-6 decreased gradually as the incubation time in plasma increased. However, the activity was not completely inhibited even after 24 h of exposure. This suggests that incubation in plasma does not entirely suppress the antifungal effects of blap-6, indicating a reasonable degree of plasma stability for this compound. 

The effect of protease on blap-6 was detected by HPLC. When blap-6 was incubated with trypsin for 1 h, blap-6 began to degrade, and the half-time (t_1/2_) was about 6 h. When blap-6 was incubated with chymotrypsin for 1 h, the degradation rate was 19%, and the half-time (t_1/2_) was about 5 h. With the increase in incubation time, the degradation rate of blap-6 gradually increased, and the degradation rate reached 90% at 24 h (Figure 8B and Figure S1), indicating that protease may affect the activity of blap-6.

## 3. Discussion

In recent years, the growing application of bioinformatics in medicine has created new avenues for research and drug discovery. For example, AI-driven bioinformatics techniques have become increasingly important in biomedical fields such as drug discovery, medical image analysis, and network biology [47,48]. In this study, we leveraged the deep learning method to identify many antimicrobial peptides from the hemolymph transcriptome of *B. rhynchopetera.* Traditionally, the search for proteins with homologous sequences or similar functions has relied on techniques such as BLAST or using hidden Markov models to identify conserved motifs and domains [49,50,51]. However, these methods can be challenging to apply to shorter peptides, especially those lacking significant sequence homology [52]. Our study overcame this limitation by treating the peptide sequences as textual data and using deep learning to discover novel antimicrobial peptides.

In the past few decades, AMPs have been developed as a promising alternative to antibiotics and are key peptides in treatment and drug discovery. There are hundreds of AMPs identified by virtual or experimental analysis. However, AMPs encounter inherent limitations in the development of clinical drugs, such as hemolytic activity, cytotoxicity, poor metabolic stability, low bioavailability, rapid enzymatic degradation, and drug delivery problems. To overcome these limitations, researchers have developed different strategies to overcome these shortcomings, such as chemical modification of peptides, including cyclization modification [53], aminoacylation and carboxyl amidation modification [54], alkylation of amide nitrogen [55], side-chain modifications [56], etc. If the above problems occur in the subsequent in vivo experiments, we can modify the structure of blap-6 to give full play to its advantages.

The mortality rate caused by fungal diseases is increasing, usually accompanied by the emergence of a fungal resistance spectrum, resulting in an increasing trend of fungal resistance year by year. The causes of drug resistance in fungi mainly include mutation or overexpression of drug targets, expression of efflux systems, degradation of drugs themselves, and pleiotropic drug reactions [57,58,59]. Therefore, the measures taken for the possible drug resistance of blap-6 mainly include: (1) pharmacokinetics/pharmacodynamics combined with regular monitoring of blap-6 therapeutic drugs to optimize the dose, maximize the therapeutic potential, and minimize the evolution of drug resistance while minimizing adverse reactions. (2) blap-6 combined with other antibiotics for antimicrobial therapy; (3) at the same time, control the agricultural environment and clinical fungal diseases.

Hemolymph, the immune fluid of insects, plays a crucial role in transporting nutrients, facilitating intercellular communication, and defending against pathogens. When insects are stimulated or infected, they secrete antimicrobial peptides into the hemolymph as a first line of immune defense in response to systemic pathogens [60,61]. As the first line of defense against microbial invasion, AMPs play an extremely important role [62]. The antimicrobial peptide blap-6, identified in the hemolymph of *B. rhynchopetera*, may contribute to the insect’s molecular defense mechanisms and adaptation to its environment. These findings lay the groundwork for further investigation into the physiological and ecological roles of antimicrobial peptides in *B. rhynchopetera*.

Although blap-6 has no sequence similarity with other insect defensins, the peptide exhibits strong antimicrobial activity, suggesting that it may be related in terms of amino acid distribution and charge. In addition, we found that blap-6 is rich in arginine (Arg), which accounts for 41.2% of the total amino acid content. It also contains the hydrophobic amino acid tryptophan (Trp). Arg and Trp are the two natural residues most closely related to AMP activity, probably because they are the most hydrophobic or cationic residues, respectively [63]. This provides a basis for the amphiphilic antibacterial activity of blap-6.

*C. neoformans* invade the host through the human respiratory system, especially in people with weakened immune systems., such as organ transplant recipients, HIV/AIDS patients, and those undergoing chemotherapy treatment. In these immunocompromised individuals, the infection can lead to central nervous system trauma and the onset of cryptococcal meningitis [64,65]. Unfortunately, the existing treatment options for *C. neoformans* infection are limited. The currently available antifungal drugs are hampered by toxicity concerns and the increasing prevalence of drug-resistant strains. This situation urgently calls for the development of new antifungal agents that can effectively treat the fungus without substantial side effects. AMPs have emerged as promising alternatives to the currently available antimicrobials. AMPs possess broad-spectrum antimicrobial activity and appear to have fewer adverse effects compared to conventional antibiotics. Examples of AMPs that are now clinically being used include cyclic peptides (lysozyme, catanocin, and pristinamycin), glycopeptides (vancomycin, telavanxin, and ramopram), and lipopeptides (daptomycin and colistin) [66,67]. The present study focuses on a novel antimicrobial peptide, blap-6, derived from the Chinese folk medicinal insect *B. rhynchopetera*. Furthermore, the website server (http://pmlabstack.pythonanywhere.com/SCMB3PP, accessed on 30 April 2024) is established to identify novel and potential blood–brain barrier penetrating peptides (B3PPs) [68], and the result indicated that blap-6 was B3PPs when choosing model type Dataset-2, and the score (Threshold = 206) was 350.88. Here, we have systematically investigated its antimicrobial activity, pharmacological properties, and mechanism of action against *C. neoformans*.

The MIC assay provides a visual assessment of the antibacterial activity of the polypeptide, allowing us to evaluate the potential development prospects of the screened polypeptide. In addition to antibacterial activity, blap-6 also exhibits antifungal properties. The most significant effect was observed against the *C. neoformans* ATCC 32045 strain, with a MIC value that is 10–125 times lower than the MICs against other bacteria and fungi tested.

Currently reported antimicrobial peptides (AMPs) with anti-*C. neoformans* activity include DvAMP [69], PrAMP [70], and AMP-17 [71], and their MIC values are higher than 1 μM against this fungal pathogen. In contrast, the antimicrobial peptide blap-6 identified in this study has a MIC of 0.81 μM against *C. neoformans*. Although the MIC of blap-6 is still higher than that of the antifungal drug amphotericin B, blap-6 holds promise as a potential antifungal agent due to the concerning side effects associated with amphotericin B, such as kidney and liver damage. Notably, compared to the positive control group fluconazole, blap-6 was able to kill *C. neoformans* ATCC 32045 within 60 min, indicating a faster fungicidal activity than fluconazole. This may be attributed to the fact that blap-6 acts on the *C. neoformans* cell membrane, leading to rapid cell damage and death. 

The results of TEM analysis showed that the cell wall of *C. neoformans* became thinner, the plasma membrane collapsed, integrity was lost, and the cytoplasmic content coagulated. The membrane potential experiment showed that the membrane potential of *C. neoformans* treated with blap-6 was hyperpolarized, and it was further inferred that the antibacterial activity of blap-6 was related to the destruction of the fungal cell membrane.

Biofilm is a protective growth pattern formed by microorganisms during long-term evolution. These microbial communities can adhere to surfaces, forming a strong barrier that is highly resistant to antibiotics and immune defense mechanisms, contributing to chronic and recalcitrant infectious diseases. The results of this study showed that blap-6 could significantly inhibit the formation of biofilm at a concentration of 1 × MIC. In addition, blap-6 had a significant scavenging effect on the pre-formed 48 h *C. neoformans* biofilm, which was dose-dependent. This ability of blap-6 to attach biofilms, compared to traditional antibiotics, suggests its potential for application in a wider range of scenarios. 

In addition to evaluating the antimicrobial efficacy of peptides, assessing their in vitro safety is a prerequisite for further consideration as potential clinical drug candidates. In the human red blood cell hemolysis test, the polypeptide did not show significant hemolytic activity up to a concentration of 83.3 μM (Figure 7A). Similarly, in the cytotoxicity experiment, the polypeptide blap-6 showed no obvious toxicity to mouse macrophage Raw 264.7 cells up to 166.7 μM, although some cytotoxicity was observed at higher concentrations (Figure 7B). However, considering that the MIC value of blap-6 is 0.81 μM (Table 2), which is much lower than its cytotoxic concentration, its safety is also guaranteed under the premise of ensuring its activity. 

The results presented here strongly suggest that blap-6 has potential applications as a novel antifungal peptide. *Cryptococcus*, particularly *C. neoformans*, is a frequent causative agent of various human infections, including pneumonia and meningitis. The broad-spectrum antifungal activity of blap-6 observed in this study and its potential for treating cryptococcosis open up new avenues for therapeutic development. Furthermore, we anticipate that continued bioinformatics analyses of the gland transcriptome of *B. rhynchopetera* will unveil additional potent antimicrobial peptides with therapeutic applications.

## 4. Materials and Methods

### 4.1. Collection of Hemolymph of B. rhynchopetera and Transcriptome Sequencing 

These adults of *B. rhynchopetera* were collected from Yuanmou County, Chuxiong autonomous prefecture, in Yunnan Province, China. Then, these adults were dissected quickly, and the tissues of the hemolymph were quickly put into liquid nitrogen. The samples were paired-end sequenced on the Illumina platform, and 150 bp paired-end reads were generated at Novogene Bioinformatics Technology Co., Ltd. (Beijing, China). To produce CDS protein sequences and high-quality transcripts, a set of specialized Perl scripts was used to process the raw data.

### 4.2. Antimicrobial Peptide Identification, Prediction, and Synthesis

High-quality transcriptome data were processed further using Tbtools software [72]. Positively charged amino acid-rich peptides were predicted and examined, and the resulting sequences were uploaded to the Antimicrobial Peptide Scanner vr.2 (https://dveltri.com/ascan/v2/ascan.html, accessed on 5 April 2023) for high-throughput virtual screening by the deep learning method [73]. The antimicrobial peptide sequences with the highest score were finally chosen. The ExPASy website (http://www.expasy.org/tools/, accessed on 5 April 2023) was utilized to assess the physicochemical characteristics of every peptide. All the peptides were synthesized by Go Top Peptide Biotech Ltd. (Hangzhou, China), and the purity of these peptides is required to be higher than 95% in reversed-phase high-performance liquid chromatography (RP-HPLC) and mass spectrometry (MS) [74].

### 4.3. Structure Prediction

The HeliQuest (https://heliquest.ipmc.cnrs.fr, accessed on 5 April 2023) website was used to analyze the hydrophobic properties and the peptide backbone of blap-6; the NPS @ website (https://npsa-prabi.ibcp.fr/cgi-bin/npsa_automat.pl?page=/NPSA/npsa_sopma.html, accessed on 5 April 2023) was used to predict the two-dimensional structure of blap-6; and the NCBI website (https://www.ncbi.nlm.nih.gov, accessed on 5 April 2023) was predicted to have no homologous sequence for blap-6.

The secondary structure of blap-6 in different solvent environments was detected by circular dichroism (CD, J-1500, JASCO, Kyoto, Japan) under the condition of nitrogen purge at 298 K. The broad spectrum was the accumulation of three scans measured in 0 mM, 4 mM, and 8 mM sodium dodecyl sulfate (SDS, Biofoxx, Germany) solutions, respectively. The final concentration of blap-6 was 208 μM (0.5 mg/mL). The scanning wavelength range was 190–270 nm, and the average scanning rate was 100 nm/min. The spectral bandwidth is 0.2 nm. The light diameter of the cuvette was 1 mm. All broad-spectrum samples were collected three times, and the average value was calculated. Finally, the CD data were smoothed by Spectra Manager™ Suite software (version Spectra Manager 2.15), saved, and uploaded to the DichroWeb website (http://dichroweb.cryst.bbk.ac.uk, accessed on 5 April 2023). The wave number range was selected from 190 to 240 nm, and the relative content of the secondary structure was evaluated by the CONTIN algorithm for verification.

### 4.4. Sequence Alignment and Phylogenetic Analysis

An alignment homology search of blap-6 was performed on the national center for Blast website (https://blast.ncbi.nlm.nih.gov/Blast.cgi, accessed on 5 April 2023). The sequences were aligned using Clustal W (https://www.genome.jp/tools-bin/clustalw, accessed on 5 April 2023) and embellished using Escript 3.0 (https://espript.ibcp.fr/ESPript/cgi-bin/ESPript.cgi, accessed on 5 April 2023). Phylogenetic analysis was performed using Mega software (version 11.0) for the maximum likelihood tree.

### 4.5. Bacteria and Fungi Preparation and Growth Condition

These standard strains of *Escherichia coli* (*E. coli*, ATCC 8739), *Staphylococcus aureus* (*S. aureus*, ATCC 6538), *Pseudomonas aeruginosa* (*P. aeruginosa*, ATCC 27853), *Acinetobacter baumannii* (*A. baumannii*, ATCC 1968), *Candida albicans* (ATCC 10231), and the clinical strain of Methicillin-resistant *Staphylococcus aureus* (MRSA, MRSA-Z) were obtained from Kunming Medical University. The standard strain of *C. neoformans* (ATCC 32045) was purchased from Shanghai Yingxin Laboratory Co., Ltd. (CAS NO. YXJ50058). Different strains of *E. coli*, *S. aureus*, MRSA, *P. aeruginosa*, and *A. baumannii* were cultured with shaking at 37 °C in Luria–Bertani (LB) medium. *C. neoformans* was cultured at 35 °C in Sabouraud Dextrose Agar (SDA) medium according to the manufacturer’s instructions.

### 4.6. Determination of the Minimum Inhibitory Concentration 

According to Clinical and Laboratory Standards Institute (CLSI) protocols, the minimum inhibitory concentration (MIC) is the minimum drug concentration at which antibiotics inhibit the growth and reproduction of strains [75]. The different strains were grown in their corresponding media at 35 °C or 37 °C until they entered the exponential growth phase. Fluconazole and amphotericin B were used as positive controls, and sterile 0.9% NaCl was used as a negative control. The peptides were diluted to various concentrations in sterile saline. According to 1 OD = 1 × 10^9^ CFU/mL, the above bacterial solution was diluted to 2 × 10^5^ CFU/mL with the corresponding medium. In advance, 100 μL of normal saline was added to a sterile 96-well plate, and 200 μL of the sample to be tested was added to the first well. The sample to be tested was subjected to double gradient dilution, and then 100 μL of bacterial solution with a concentration of 2 × 10^5^ was added to each well. It was cultured at 35 °C or 37 °C for different times. The light absorption value of the bacterial solution at 600 nm was detected by a microplate reader (Epoch Etock, Biotek, Winooski, VT, USA), and the average concentration of the samples in the pores and adjacent pores that could not detect microbial growth was used as the MIC value.

### 4.7. Transmission Electron Microscope (TEM)

The suspension of *C. neoformans* ATCC 32045 was prepared and then centrifuged at 3500 rpm for 5 min. The supernatant was discarded, and sterile normal saline was added for washing. That is, the above operation was repeated three times and re-suspended. The blap-6 was added to the above fungal solution, and the final concentration was 1 × MIC (0.81 μM). The positive control, including the concentration of fluconazole and amphotericin, was 1 × MIC. The negative control was added with the same volume of sterile normal saline. After the sample was added to the fungal solution, it was incubated in an incubator at 37 °C for 12 h, and then centrifuged at 1000 rpm for 10 min. The supernatant was discarded, and osmium tetroxide was slowly added along the tube wall for fixation and fixed at 4 °C for 3 h. Gradient ethanol was used for dehydration, infiltration, and embedding. Finally, ultrathin sections were performed. After the above operation, the morphological changes in *C. neoformans* were observed by TEM (Tecnai G2 Spirit, FEI, Eindhoven, Holland) and photographed.

### 4.8. Fungal-Killing Kinetics 

The single colony of *C. neoformans* ATCC 32045 was selected and cultured at 180 rpm and 37 °C, washed three times with normal saline, and then resuspended, and the fungal solution was diluted to 1 × 10^6^ CFU/mL with SDB medium. The positive control was fluconazole, and the negative control was normal saline. The final concentrations of fluconazole and blap-6 were 1 × MIC, 5 × MIC, and 10 × MIC, respectively. The fungal liquid after adding the sample was quickly put into the 37 °C constant temperature incubator, and the shaking culture was carried out at 180 rpm. The above fungal liquid (10 μL) was diluted 100 times with normal saline at eight time points (0 min, 10 min, 30 min, 60 min, 180 min, 360 min, 720 min, and 1440 min), and then 100 μL of fungal liquid was coated with SDA medium. The plate was placed in a constant temperature incubator for inverted culture for 72 h at 37 °C, and then the single colony on the plate was counted.

### 4.9. Biofilm Inhibition

The single colony of *C. neoformans* ATCC 32045 was selected and cultured at 180 rpm and 37 °C. The physiological saline was washed three times and then re-suspended. The fungal concentration was adjusted to 1 × 10^6^ CFU/mL, and then 100 μL of the fungal solution was added to each hole in the 96-well plate. The samples to be tested were diluted with sterile saline to 0.5×, 1×, 2×, 4×, and 8 × MIC, and 100 μL was added to the 96-well plate and mixed well. Three replicate wells were set for each concentration. The negative control group was added to the same volume of sterile saline. The positive control group was added with 10 μM fluconazole and amphotericin B, respectively, and the above group was incubated in a 37 °C incubator for 48 h. After that, the supernatant was discarded and washed with sterile PBS three times, 1 min each time. And then, 99% methanol was added and fixed for 5 min. After staining with 100 μL 0.1% crystal violet for 5 min, the plate was washed with PBS, and 100 μL 95% ethanol was added. The absorbance at 600 nm was detected by a microplate reader.

### 4.10. Biofilm Eradication

The single colony of *C. neoformans* ATCC 32045 was picked and cultured at 180 rpm and 37 °C. After washing with normal saline three times and being resuspended, the concentration of the fungal solution was adjusted to 1 × 10^6^ CFU/mL and then added to a 96-well plate. Each well was incubated at 37 °C for 48 h. The fungal solution was discarded and washed three times with PBS for 1 min each time. The sample to be tested was diluted with normal saline to 0.5×, 1×, 2×, 4× and 8 × MIC, and 200 μL was added to the 96-well plate, respectively. Each concentration was set to three replicate wells, and the negative control group was added with the same volume of sterile normal saline. In the positive control group, 10 μM fluconazole and 10 μM amphotericin B were added and incubated at 37 °C for 48 h. After that, the supernatant was discarded and washed three times with sterile PBS for 1 min each time. And then, 99% methanol was added and fixed for 5 min. Methanol was removed and dried under the ultra-clean bench. After staining with 100 μL 0.1% crystal violet for 5 min, wash the plate with PBS, dry, and add 100 μL 95% ethanol. The absorbance at 600 nm was detected by a microplate reader.

### 4.11. Two-Photon Laser Scanning Microscope (TPLSM)

A single colony of *C. neoformans* ATCC 32045 was selected and cultured at 180 rpm at 37 °C. The suspension was washed three times with normal saline and then resuspended. The concentration of the suspension was adjusted to 1 × 10^6^ CFU/mL, and then 500 μL was added to each hole in the 24-well plate. The pellets were incubated at 37 °C for 48 h, the supernatant was discarded, washed three times with PBS, and the sample to be tested was diluted with sterile normal saline to 0.5×, 1×, 2×, 4×, 8 × MIC, and 500 μL was added to the 96-well plate, respectively. The negative control group was added to the same volume of sterile normal saline. In the positive control group, 10 μM fluconazole and amphotericin B were added and incubated at 37 °C for 48 h. The non-adherent cells were washed with PBS, and then 500 μL of the FDA (Solarbio, Beijing, China) and PI (Solarbio, Beijing, China) mixture was added and co-stained in the dark for 30 min. The FDA content in the mixture was 10 μg/mL, and the PI content was 5 μg/mL. The two-photon laser scanning microscope (TPLSM, A1MP+, Nikon, Tokyo, Japan) was used to observe. The 488 nm and 555 nm excitations of FDA and PI were imaged separately. Due to the hydrolysis of FDA by living cells, green fluorescence was accumulated. The dead cells were marked red by PI, so the cell viability could be measured and analyzed by Image J.

### 4.12. Membrane Potential 

DiSC3(5) is a fluorescent probe often used as a tracer dye to assess the cell membrane potential [76]. Therefore, we assessed the effects of blap-6 on the cell membrane potential changes in *C. neoformans* ATCC 32045 using DiSC3(5) staining. *C. neoformans* ATCC 32045 was selected for SDB medium and cultured in a constant-temperature shaker at 35 °C to the logarithmic phase. The supernatant was removed by centrifugation at 3500 rpm for 5 min, washed three times with normal saline, and then the fungal solution was diluted with normal saline containing 100 mM potassium chloride to an OD_600_ of about 0.1. DiSC3(5) (AAT Bioquest, Pleasanton, CA, USA) dye was dissolved in DMSO and diluted with 1% DMSO, so that the final concentration of DiSC3(5) added to the fungi solution was 0.5 μM. 

The above-mixed suspension was added to a fluorescence-specific 96-well plate, 190 μL per well, and the excitation/emission wavelength was set at 622/670 nm, and a microplate reader was used for measurement. The measurement was performed until the microplate reader value was stable and then continued for 20 min. The experimental group was added with 10 μL blap-6 to make the final concentrations 16 × MIC, 8 × MIC, and 1 × MIC, respectively. The positive control group was added with 10 μL of valinomycin to a final concentration of 10 μM. The negative control was added to 10 μL of normal saline. Continue to measure for 60 min. Three replicates were set up in the experiment.

### 4.13. Reactive Oxygen Species (ROS) Detection

2′,7′-Dichlorofluorescin diacetate (H2DCFDA) is a cell-permeable probe for detecting intracellular ROS [77]. *C. neoformans* ATCC 32045 was selected for SDB medium, cultured at 35 °C to logarithmic phase, centrifuged at 8000 rpm for 5 min to remove the supernatant, washed with normal saline three times, and then diluted the fungal solution to OD_600_ = 0.1. The different concentrations of blap-6 (1 × MIC, 8 × MIC, and 32 × MIC) were incubated with the diluted fungal solution at 35 °C for 30 min, and 0.1 mM H_2_O_2_ was used as a positive control. The negative control group was treated with normal saline and incubated at 37 °C for 30 min. Centrifuged at 8000 rpm and 4 °C for 5 min, discard the supernatant, leaving the cell precipitate. The H2DCFDA dye (Boer, Shanghai, China) was dissolved in DMSO and diluted with normal saline containing 1% DMSO to a final concentration of 10 μM. And added 1 mL of H2DCFDA (10 μM) working solution to each tube and incubated at 35 °C for 30 min. And then precipitated the pellets, discarded the supernatant, and paid attention not to suck out the fungi. The fungi were washed again with normal saline to remove H2DCFDA that did not enter the fungi. The excitation/wavelength emission was set at 488/525 nm, and three replicates were set in the experiment. 

### 4.14. Hemolytic Activity

The healthy human blood was collected in an anticoagulant tube containing sodium citrate at 3500 rpm and centrifuged for 5 min. The supernatant was discarded, and the red blood cells were washed 3–5 times with normal saline until the supernatant was no longer red after centrifugation. The concentration of the washed red blood cells was adjusted to 1 × 10^7^–10^8^ cells/mL by using an appropriate amount of normal saline. The samples to be tested were prepared into different concentration gradients (1.3, 2.6, 5.2, 10.4, 20.8, 41.7, 83.3, and 166.6 μM), 100 μL was added to each hole, and three replicates were set. Add 100 μL of red blood cell suspension in each hole, incubate at 37 °C in an incubator for 30 min, then centrifuge at 1500 rpm for 5 min. The supernatant was collected, and the light absorption value at 540 nm was detected by a microplate reader. In this experiment, sterile saline was used as a negative control, and the same volume of 10% Triton X-100 was used as a positive control. The hemolytic activity was proportional to the light absorption value at 540 nm, and the hemolysis rate was calculated using the following formula: hemolysis rate (%) = (hemolysis of experimental group-negative control)/(hemolysis of positive control-negative control) ×100%. 

### 4.15. Cell Cytotoxicity

Cytotoxicity was detected in human HEK293 embryonic cells purchased from the Kunming Institute of Zoology. The cell line was cultured in Dulbecco’s modified Eagle’s medium (DMEM, Gibco, Billings, MT, USA) medium containing 10% fetal bovine serum and 1% penicillin-streptomycin at 37 °C and 5% CO_2_, respectively. When the cells covered 80% of the bottom of the bottle, the cell concentration was adjusted to 1 × 10^6^ cells/mL with the medium, and 100 μL of the above cell suspension was placed in a sterile 96-well plate. The cells were cultured in an incubator for 24 h, and 100 μL of different concentrations of samples to be tested were added. The concentration gradient was set to 0.6, 1.3, 2.6, 5.2, 10.4, 20.8, 41.7, 83.3, and 166.6 μM, and three parallel holes were set for each concentration. The blank control was added with the same volume of sterile saline. The cells were cultured in a constant temperature incubator at 37 °C and 5% CO_2_ for 24 h, and 10 μL of Cell Counting Kit-8 (CCK-8, MCE, Princeton, NJ, USA) solution was added to each hole. The cells were incubated in the incubator for 1 h under dark conditions, and the light absorption value of each hole at 450 nm was detected by a microplate reader. The cell survival rate was calculated according to the manufacturer’s instructions.

### 4.16. Plasma Stability 

The obtained human blood was placed in an anticoagulant tube containing sodium citrate, centrifuged at 3500 rpm for 10 min, and the yellow supernatant was carefully sucked. The obtained plasma was doubled with sterile saline, and the polypeptide blap-6 was added. The final concentration of blap-6 was 10 mg/mL. Subsequently, the plasma dissolved in blap-6 was incubated in a 37 °C constant temperature incubator, and 10 μL was taken at 0, 1, 2, 4, 6, 8, 12, and 24 h time points, respectively. The antifungal activity of blap-6 against *C. neoformans* was detected by the inhibition zone method after incubation with plasma, and three replicates were set at each time point.

### 4.17. The Effects of Protease on Blap-6

For investigating the impact of protease on blap-6, trypsin and chymotrypsin were chosen as the enzymes to be studied in the experiment. A total of 2.5 mg/mL proteases (including trypsin or chymotrypsin) and 2.5 mg/mL of blap-6 were mixed at a ratio of 1:40 and incubated at 35 °C for 0 h, 1 h, 3 h, 6 h, 12 h, 24 h, and 48 h, respectively. Then these samples (including blap-6 as a standard) were detected by high-performance liquid chromatography (HPLC, Waters, Milford, MA, USA) on a C18 column (4.6 mm × 250 mm, Waters, USA). Solvent A was ultrapure water, and solvent B was acetonitrile. The flow rate was 0.5 mL/min, and the detection wavelength was 280 nm. The column was balanced with 95% solvent A in 0–3 min and eluted with 95–40% solvent A in 3–10 min, 40–5% solvent A in 10–15 min, and 5–95% solvent A in 15–20 min. The degradation rate was calculated by the peak area of the HPLC.

### 4.18. Statistical Analysis

The data analysis was conducted utilizing GraphPad Prism 6.01 (GraphPad Software, San Diego, CA, USA). Statistical differences were assessed using a one-way ANOVA. The data are presented as mean ± standard deviation (mean ± SD), with statistical significance defined as * *p* < 0.05, ** *p* < 0.01.

## 5. Conclusions

In conclusion, this study describes the structure and antimicrobial activity of blap-6, a peptide from the hemolymph of *B. rhynchopetera*, an insect used in Chinese folk medicine. Blap-6 showed high antifungal activity against *C. neoformans*. In vitro experiments showed that blap-6 had high plasma stability and fewer side effects, including low cytotoxicity and hemolytic activity. The present findings suggest that blap-6 may be a candidate drug for the treatment of human pathogenic fungi. Future studies need to verify the therapeutic effect of blap-6 in vivo by developing models of meningitis.

## Figures and Tables

**Table 1:** Physicochemical properties of different peptides.

Peptides	Sequence	Length	Nc	Mw	GRAVY	Pr (n/%)	Nr (n/%)
blap-1	YSKPTRWANFLMTLYPTICHITKVTLS	27	3.1	3185.79	0.104	12/44.44	15/55.56
blap-2	VILFHVACWIILLQLIRNFSSRRHGHGFFYIFSAA	35	3.3	4133.92	0.929	13/37.14	22/62.86
blap-3	PLRPSQRYWRGSKGPNGRVLYNIFHIRLRKIIKN	34	9.1	4134.90	−0.900	19/55.88	15/44.12
blap-4	NTTPFYLFFLSGATGKFYYFWKVYFFLNTAAYHKS	35	3.1	4241.86	0.129	14/40.00	21/60.00
blap-5	IFIVLLFCLLRWGKRYTFSNTNRYWYPLILTKS	33	5.0	4126.96	0.355	13/39.39	20/60.61
blap-6	KRCRFRIYRWGFPRRRF	17	8.0	2400.89	−1.42	9/52.94	8/47.06
blap-7	KRALSLPKMREDRLLYRGRA	20	5.0	2429.92	−1.035	11/55.0	9/45.0
blap-8	RWKERKKWQKRWKRKKG	17	10.0	2412.92	−3.259	14/82.35	3/17.65
blap-9	FIKKMLGNFSNYVRRPGKR	19	6.0	2311.78	−0.879	11/57.89	8/42.11
blap-10	RWNTSRWLRL	10	3.0	1387.61	−1.270	6/60.00	4/40.00
blap-11	PFVIFSNFLIGFIVRVVKLISPGKYYLSG	29	3.0	3274.98	1.110	10/34.48	19/65.52

Nc: Net charge; Mw: Molecular weight; GRAVY: Grand average of hydropathicity (“−” represents hydrophobic peptides); Pr: Polar residues; Nr: Non-polar residues.

**Table 2:** MICs of different AMPs against different microorganism strains (μM).

Peptides	*E. coli*	*S. aureus*	*A. baumannii*	*P. aeruginosa*	MRSA	*C. albicans*	*C. neoformans*
blap-1	NA	NA	NA	NA	NA	NA	NA
blap-2	NA	NA	NA	NA	NA	NA	NA
blap-3	NA	NA	NA	NA	NA	NA	NA
blap-4	NA	NA	NA	NA	NA	NA	NA
blap-5	NA	NA	NA	NA	NA	NA	NA
blap-6	9.77	9.77	19.54	9.70	4.89	1.22	0.81
blap-7	NA	NA	NA	NA	NA	156.30	312.50
blap-8	312.50	156.3	321.50	7.81	156.30	19.53	19.53
blap-9	81.10	81.10	81.10	81.10	81.10	40.55	81.10
blap-10	NA	NA	NA	312.50	19.54	156.30	312.5
blap-11	NA	NA	NA	NA	NA	NA	NA
Ampicillin	31.56	63.12	31.56	31.56	126.24	NA	NA
Fluconazole	NA	NA	NA	NA	NA	0.61	6.36
Amphotericin B	NA	NA	NA	NA	NA	0.05	0.05

NA: no antibacterial or antifungal activity. These values represent the mean of three independent experiments.

## Data Availability

The data used to support the findings of this study are available upon request from the corresponding author.

## Supplementary Materials

The supporting information can be downloaded at: https://www.mdpi.com/article/10.3390/ijms25105336/s1.
