# Supplementary material for: Blap-6, a Novel Antifungal Peptide from the Chinese Medicinal Beetle Blaps rhynchopetera against Cryptococcus neoformans
Source: Int J Mol Sci. 2024 May 14;25(10):5336. doi: 10.3390/ijms25105336 (PMC11121495; doi:10.3390/ijms25105336)
Supplement: Supplementary file 1 [file ijms-25-05336-s001.zip › ijms-2993866-supplementary.pdf]

Supplementary information

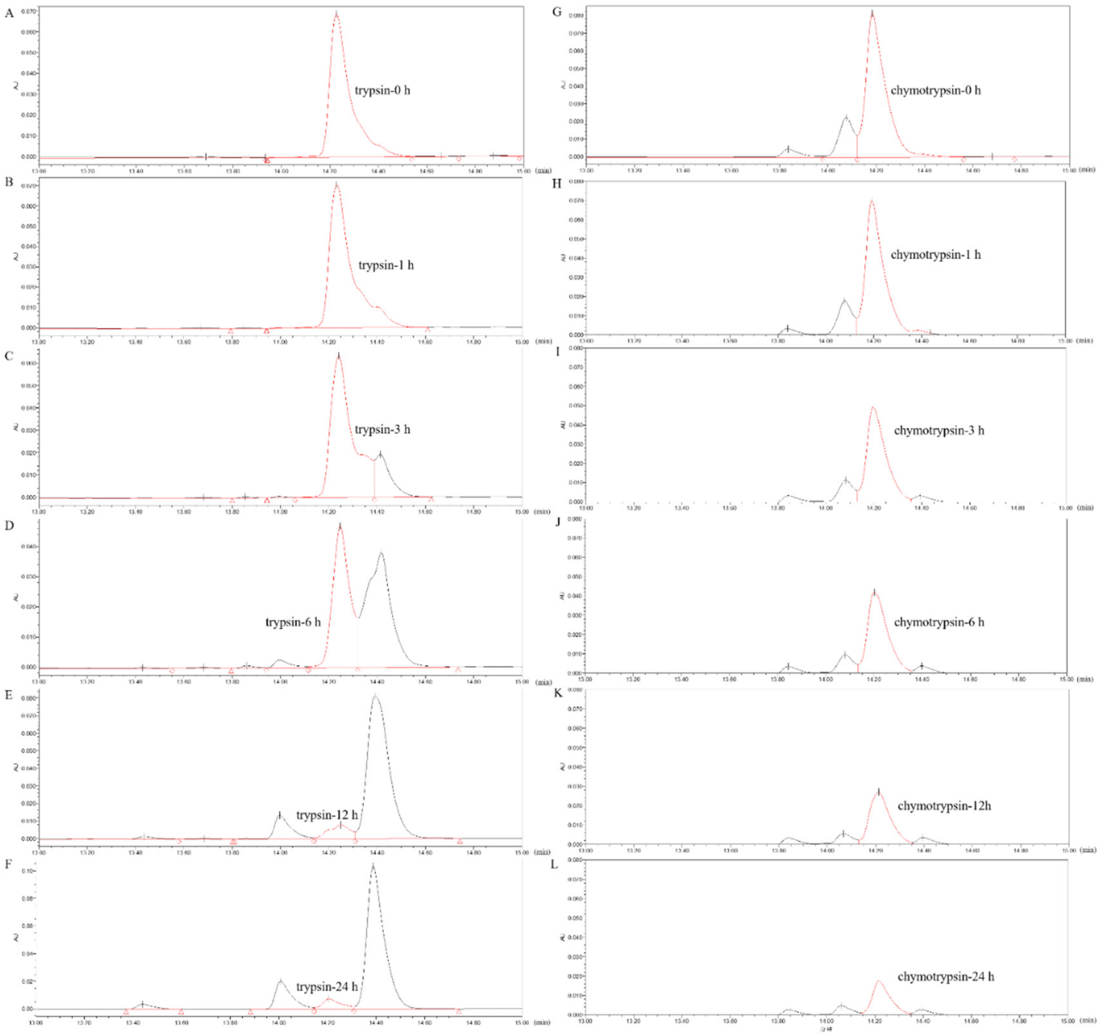

Figure S1. The effect of proteases (including trypsin and chymotrypsin) on the blap-6 was detected by high-performance liquid chromatography (HPLC) on a C18 column (4.6 mm × 250 mm, Waters, USA). (A) Blap-6 was incubated with trypsin for 0 h. (B) Blap-6 was incubated with trypsin for 1 h. (C) Blap-6 was incubated with trypsin for 3 h. (D) Blap-6 was incubated with trypsin for 6 h. (E) Blap-6 was incubated with trypsin for 12 h. (F) Blap-6 was incubated with trypsin for 24 h. (G) Blap-6 was incubated with chymotrypsin for 0 h. (H) Blap-6 was incubated with chymotrypsin for 1 h. (I) Blap-6 was incubated with chymotrypsin for 3 h. (J) Blap-6 was incubated with chymotrypsin for 6 h. (K) Blap-6 was incubated with chymotrypsin for 12 h. (L) Blap-6 was incubated with chymotrypsin for 24 h.
